# Sequential drug delivery by injectable macroporous hydrogels for combined photodynamic-chemotherapy

**DOI:** 10.1186/s12951-021-01066-1

**Published:** 2021-10-23

**Authors:** Yuanyuan Zhong, Li Zhang, Shian Sun, Zhenghao Zhou, Yunsu Ma, Hao Hong, Dongzhi Yang

**Affiliations:** 1 Jiangsu Key Laboratory of New Drug Research and Clinical Pharmacy, Xuzhou Medical University, Xuzhou, 221004 Jiangsu China; 2 Medical School, Nanjing University, Nanjing, 210093 Jiangsu China; 3 Xuzhou Air Force College, Xuzhou, 221000 Jiangsu China

**Keywords:** Sequential drug delivery, Combretastatin A4 phosphate, Doxorubicin, Photodynamic therapy, Combined photodynamic-chemotherapy

## Abstract

With hollow mesoporous silica (hMSN) and injectable macroporous hydrogel (Gel) used as the internal and external drug-loading material respectively, a sequential drug delivery system DOX-CA4P@Gel was constructed, in which combretastatin A4 phosphate (CA4P) and doxorubicin (DOX) were both loaded. The anti-angiogenic drug, CA4P was initially released due to the degradation of Gel, followed by the anti-cell proliferative drug, DOX, released from hMSN in tumor microenvironment. Results showed that CA4P was mainly released at the early stage. At 48 h, CA4P release reached 71.08%, while DOX was only 24.39%. At 144 h, CA4P was 78.20%, while DOX release significantly increased to 61.60%, showing an obvious sequential release behavior. Photodynamic properties of porphyrin endow hydrogel (*ϕ*_Δ(Gel)_ = 0.91) with enhanced tumor therapy effect. In vitro and in vivo experiments showed that dual drugs treated groups have better tumor inhibition than solo drug under near infrared laser irradiation, indicating the effectivity of combined photodynamic-chemotherapy.

**Supplementary Information:**

The online version contains supplementary material available at 10.1186/s12951-021-01066-1.

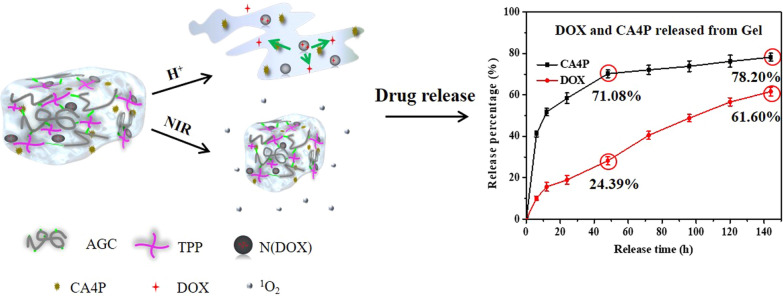

## Introduction

Cancer is still one of the significant threats to human health. Current cancer therapies, such as chemotherapy [1], phototherapy [2], hyperthermia [3] and radiotherapy [4], are widely applied but they have their own limited properties or unavoidable side effects. For example, patients always suffer from serious side effects by intrinsic off-target toxicity of chemotherapy drugs, also endure the possible low effective treatment for the limited relatively short resident time in tumor cells [5]. Phototherapy and hyperthermia against deep tumors may greatly limited by insufficient luminous flux [6]. Radiotherapy can also damage nearby healthy cells beside killing cancer cells [7]. Especially for chemotherapy, the drug’s resident time in tumor cells is relatively short, which limited the theragnostic effect. Therefore, it’s necessary to develop local sequential delivery of multiple drugs for optimal efficacy, potency, and safety. The co-administration of dual drugs with different physicochemical properties and specific administration sequence is very important to overcome drug resistance and reduce side effects in tumor therapy. Anti-cell proliferative drugs, such as doxorubicin (DOX) and paclitaxel (PTX), can kill cancer cells from different proliferation stages. However, due to the support of peripheral blood vessels, the survived tumor cells could still obtain nutrition supply and continue to proliferate [8]. Anti-angiogenic drugs can specifically target vascular growth factors to inhibit the establishment of peri-tumoral blood vessels [9]. CA4P is one of novel angiogenesis inhibitors, which can induce apoptosis by binding tubulin dimers and preventing microtubule polymerization. It’s reported that CA4P has the potential to sensitize drug-resistant MCF-7/ADR cells to DOX due to the inhibition of angiogenesis [10]. Anti-angiogenesis combined with anti-cell proliferation has become a novel tumor therapy strategy, which was referred as “A+ strategy” [11]. When both drugs were applied to the tumor site at the right time, they could maximize the therapeutic effect. We hoped to design a system that anti-angiogenic drugs could be released firstly to inhibit the growth of new tumor vasculatures, while anti-cell proliferative drugs could be slowly released and accumulated in the tumor site to effectively kill tumor cells.

Hydrogel is composed of a large amount of water and cross-linked polymers. The abundant water content (70–99%) in hydrogels resembles biological tissues, and they usually have good biocompatibility and loading hydrophilic drugs capacity [12]. Smart hydrogels (temperature responsive [13], pH responsive [14], light responsive [15], or NO responsive [16]) were frequently used in controlled drug release studies due to their unique properties. For example, Kim et al. [17] prepared a hydrogel/microsphere composite, which could sequentially release doxorubicin (DOX) and fluorouracil (Fu) in tumors. Fu was directly encapsulated in the hydrogel, and DOX was loaded in microspheres. Results showed that 80% Fu was released at 72 h, while the release of DOX only reached about 40%, proving that the smart composite hydrogel with nano-sized microspheres could be used as a carrier for the sequential release of different drugs.

To further enhance the therapeutic effect of drugs, photodynamic therapy (PDT) has been used in combination with chemotherapy, magnetic therapy or immunity therapy because of its significant advantages such as causing less trauma, low toxicity, and palliative treatment [18]. Belali et al. [19] used amino-modified 5,10,15,20-tetraphenylporphyrin (TPP) to prepare pH-sensitive hydrogels which could achieve efficient photodynamic therapy effects. Gonzalez-Delgado et al. [20] found that hydrogels enhanced singlet oxygen generation efficiency of porphyrin derivatives when they were encapsulated in hydrogels.

Herein, we designed and prepared a novel injectable macroporous hydrogel for therapy of 4T1 breast cancer, which could load dual distinct tumor drugs (CA4P and DOX) offering a sequentially delivery profile. Furthermore, porphyrin derivatives in hydrogel trigger the formation of reactive oxygen species (ROS), achieving synergistic anticancer effects in photodynamic-chemotherapy combination therapy. The therapeutic efficacy from this combinational photodynamic/sequential chemotherapy was evaluated in vitro and in vivo.

## Experimental section

### Chemical and reagents

Tetraethyl silicate (TEOS), triethanolamine (TEA), 5,10,15,20-tetraphenylporphyrin (TPP), (5,10,15,20)-tetra(4-aminophenyl)porphyrin (NH_2_-TPP), dextran and doxorubicin hydrochloride (DOX·HCl) were all purchased from Shanghai Aladdin Biochemical Technology Co., Ltd. 3-Aminopropyltrimethoxysilane (APS) was purchased from Braunwell Chemical Technology Co., Ltd. CA4P was acquired from Solarbio Science & Technology Co., Ltd. Cetyltrimethylammonium chloride (CTAC) was purchased from Sigma-Aldrich Co., Ltd. Dulbecco’s modified Eagle’s medium (DMEM), memorial institute medium (RPMI)-1640, 3-(4,5-dimethylthiazol-2-yl)-2,5-diphenyltetrazolium bromide (MTT), 4′,6-diamidino-2-phenylindole (DAPI), 1,3-diphenylisobenzofuran (DPBF), 2′,7′-dichlorodihydrofluorescein diacetate (DCFH-DA), live/dead cell viability/toxicity kit, and Annexin V-FITC/PI staining kit were all purchased from Vicmed (Xuzhou, China). Caspase 3 polyclonal antibody (caspase 3 PAb) was purchased from Proteintech Co. (USA). PV-6001 rabbit polymer detection system was purchased from Zhongshan Jinqiao Co., Ltd. Trypsin and dimethyl sulfoxide (DMSO) were purchased from Nanjing Key Gen Biotechnology Inc. Dextran (W=70 kD), chitosan with medium viscosity (200–400 mPa·s), and sodium periodate were purchased from Macklin Biochemical Technology Co., Ltd. Other reagents were all purchased from Sinopharm Group Chemical Reagent Co., Ltd.

### Synthesis of DOX-CA4P@Gel

Hollow mesoporous silica nanoparticle (hMSN) was prepared according to our previous work [21]. Briefly, anhydrous ethanol, double distilled water and 25% ammonia (V:V:V = 180:25:4) were mixed and stirred for 10 min, followed by adding TEOS to acquire silica nanoparticles (dSiO_2_). After washing with water, dSiO_2_ was added into the mixed solution of CTAC and TEA, followed by drop-wise adding TEOS and the reaction was carried out at 80 °C for 1 h to synthesize dSiO_2_@SiO_2_.Na_2_CO_3_ powder was then added to it to allow etching dSiO_2_@SiO_2_ at 50 °C for 30 min. After washing with NaCl:methanol (1%), hMSN was achieved. Finally, hMSN was modified with amino groups on the surface via hydrolyzing APS in absolute ethanol.

Hydrogel OD/TPP was synthesized through a Schiff base reaction between the aldehyde groups from oxidized dextran (OD) and amino groups of porphyrins with the molar ratio of 1:10. Firstly, the adjacent diol groups were oxidized to aldehyde groups by sodium periodate to prepare aldehydated oxidized dextran [22]. The oxidation degree of aldehyde groups (proportion of oxidized dextran repeating units) was determined by measuring the aldehyde content via a hydroxylamine hydrochloride titration method with bromophenol blue as the indicator [23]. Secondly, 10 mg oxidized dextran (dissolved in 1 mL PBS with pH value of 6.8) was mixed with 1 mg NH_2_-TPP (dissolved in 1 mL DMF) to form OD/TPP, the unreacted OD and TPP was removed using dialysis bags (MWCO 12000–14000) against distilled water for 3 days (the water was renewed at least 5 times per day). 30 mg chitosan (dissolved in 1% acetic acid) was applied to adjust the gelation level of OD/TPP (with the mass ratio of TPP:OD:chitosan of 10:1:30) to form ‘hydrogel’. hMSN was loaded in hydrogel by simply mixing during the gelation of OD/TPP, the hydrogel loaded hMSN was referred as ‘Gel’. The excessive and unreacted reagents were removed by washing with distilled water based on the ‘no flow’ status of hydrogel.

DOX and CA4P was loaded into Gel in different methods to synthesize the composite system DOX-CA4P@Gel. Briefly, DOX·HCl aqueous solution was adjusted to pH 8.0, followed by mixing with hMSN at a mass ratio of 1:1. The mixture was stirred for 24 h at room temperature and the excess DOX was removed by dialyzing with molecular weight cut-off of 2 kDa to acquire drug-loaded microspheres DOX@hMSN [24]. The mixture of DOX@hMSN and NH_2_-TPP with a mass ratio of 1:5 was dissolved in DMF to acquire system ‘A’, and CA4P was mixed with oxidized dextran at a ratio of 1:1 (in PBS) to prepare system ‘B’. DOX-CA4P@Gel was synthesized through a Schiff base reaction between A and B. The unreacted reagents were removed by washing with distilled water.

### Characterization

The gelation time and the degradation behavior of the hydrogel under different acidic conditions were detected by an MCR 302 rheometer. Transmission electron microscopy (TEM) images were taken on a G2T12 transmission electron microscope (FEI, USA). The morphology of the hydrogel was recorded on a Q25 scanning electron microscope (FEI, USA). The hydrodynamic size and zeta potentials were measured by dynamic light scattering (DLS) (380 ZLS, NICOMP, USA). The chemical compositions were determined by X-ray photoelectron spectroscopy (XPS) (PHI Quantera SXM). All XPS spectra were calibrated by the C 1 s peak at 284.6 eV. Infrared absorption spectrum (IR) was recorded on a FTIR 8400 spectrophotometer. UV-Vis and fluorescence spectra were recorded on Hitachi U-3010 and F-4600 spectrophotometer, respectively.

### Drug release measurement

Drug release behavior was evaluated at 37 °C by respectively incubating DOX-CA4P@Gel in PBS solution with pH of 5.0, 6.4, and 7.2. CA4P release was measured by high performance liquid chromatograph (HPLC), where chromatographic separation was carried out on an Agilent Zorbax Eclipse Plus C18 column with particles size of 5.0 μm (250 × 4.6 mm) using methanol (0.01% acetic acid)-water (60:40) as the mobile phase at 1 mL/min [25]. The detection wavelength was 305 nm. DOX release was calculated by determining unbound DOX in the solution with UV-vis spectrometry [26].

### Evaluation of singlet oxygen generation efficiency

The optical property of DPBF was sensitively affected by ^1^O_2_ [27]. With DPBF as the ^1^O_2_ capture agent, the ^1^O_2_ generation efficiency of TPP in Gel can be calculated from the slopes of DPBF absorbance variation (410 nm) in the presence of photosensizers. The laser irradiation was 808 nm (0.5 W/cm^2^), with TPP in DMSO as the control, the ^1^O_2_ yield (*φ*_∆_) was calculated as follows [3]:

${\phi }_{\varDelta \left(\text{G}\text{e}\text{l}\right)}$=${\phi }_{\varDelta \left(\text{T}\text{P}\text{P}\right)}\frac{{t}_{\text{T}\text{P}\text{P}}}{{t}_{\text{G}\text{e}\text{l}}}$  Where ${\phi }_{\varDelta \left(\text{G}\text{e}\text{l}\right)}$ represents the singlet oxygen yield of Gel, ${\phi }_{\varDelta \left(\text{T}\text{P}\text{P}\right)}$was 0.52. *t*_TPP_ and *t*_Gel_ is the time for the decrease in absorption of DPBF in the presence of TPP and Gel, respectively.

### Cell culture and animal model

4T1 breast cancer cells and L929 fibroblasts cells were maintained in RPMI 1640 and DMEM medium supplemented with 10% fetal bovine serum and proper antibiotics at 37 °C with the supply of 5% CO_2_. Cells were used when they reached ~80% confluence.

All animal procedures were performed in accordance with the National Academy of Sciences’ Guidelines on the Care and Use of Laboratory Animals [28], and were approved by the Animal Ethics Committee of Xuzhou Medical University (L20210226103). Tumors were established by subcutaneous injection of 1 × 10^6^ of 4T1 cells suspended in 50 µL of PBS into female Balb/c mice (5-week-old, 18–20 g). Mice were used for therapy study when the tumor diameter reached 5–8 mm, monitored every other day by a caliper measurement.

### Fluorescence microscopy and flow cytometry

MTT assays were carried to evaluate the biocompatibility (without laser irradiation) and the therapeutic efficacy (with laser irradiation) of DOX-CA4P@Gel. DOX-CA4P@Gel at the concentration of 0.01–0.20 mg/mL were respectively incubated with 4T1 and L929 cells for 24, 48, and 72 h. MTT measurement was subsequently carried out to evaluate the cellular toxicity of DOX-CA4P@Gel [29]. To assist the study of the release behavior for CA4P and DOX, MTT assay was used to detect 4T1 cells viability under different incubation conditions, including different media with pH values of 5.0, 6.4 and 7.2, with/without laser irradiation (808 nm, 0.5 W/cm^2^, and 5 min). All experiments were performed at least three times. Five groups including CA4P@Gel, DOX@Gel, DOX-CA4P@Gel, DOX and CA4P were compared in this work.

The fluorescence microscopy was used to observe the production efficiency of ^1^O_2_ and cytotoxic effect of DOX-CA4P@Gel in 4T1 cells. 4T1 cells were cultured at a 24-well plate with concentration of 5 × 10^4^ cells/mL. After incubation with hydrogel extracted solution (0.5 mg/mL based on TPP) for 24 h with laser irradiation (808 nm, 0.5 W/cm^2^, and 5 min), the cells were stained with DCFH-DA and DAPI [30]. Cells were analyzed using fluorescence microscopy. Furthermore, the cells were also stained with live/dead staining kits to visually examine the killing effect of DOX-CA4P@Gel under laser irradiation [31].

MACSQuant 10 flow cytometry (Miltenyi Biotec, GER) was also used to study the drug release behavior of CA4P and DOX from DOX-CA4P@Gel. 4T1 cells were dispersed with a final concentration of 1 × 10^5^/well. CA4P@Gel, DOX@Gel, DOX-CA4P@Gel extracts at different concentrations were incubated with 4T1 cells for 24 h with or without laser irradiation (808 nm, 0.5 W/cm^2^, and 5 min). Flow cytometry assay was carried out after Annexin V-FITC 5 µL and PI 5 µL staining for 10 min.

### In vivo therapeutic evaluation

The mice bearing 4T1 tumor nodules were randomly divided into 10 groups (n = 8) including PBS (Control), free DOX/CA4P (mixture of 0.4 mg DOX and 1 mg CA4P), Gel, CA4P@Gel, DOX@Gel, DOX-CA4P@Gel, Gel+NIR (near infrared laser), CA4P@Gel+NIR, DOX@Gel+NIR, and DOX-CA4P@Gel+NIR. 100 µL of each sample were intravenously injected into tumor. Except for PBS and free DOX/CA4P, the concentrations of hydrogel were consistent (44 mg/mL) in eight groups. During preparation of CA4P@Gel and DOX@Gel, proper amount of DOX and CA4P were added to ensure the consistent concentration in DOX-CA4P@Gel. An 808 nm laser with 0.5 W/cm^2^ power intensity was used as the near-irradiation source. During therapy, the body weight and tumor volume (*V *= *L *× *W*^2^/2) were monitored every other day, where *L* (mm) and *W* (mm) are the long and short axes of tumor size, respectively. The tumor inhibition rate (TIR) was calculated using the equation of TIR = (*V*_c_−*V*_t_)/*V*_c_×100%, where *V*_t_ and *V*_c_ represent the tumor volumes in the treatment group and control group, respectively. After treatment, major organs including heart, liver, spleen, lung, and kidney were harvested for the hematoxylin and eosin (H&E) analysis. The 4T1 tumors harvested on day 0, 3, 7, and 14 were also frozen for histological analysis.

### Histology

Major organs were fixed in 4% formalin, transferred routinely into paraffin, sectioned into 6 μm thick slices, stained with H&E and examined by a microscopy with a magnitude of 200×. Tumors were cut into frozen slices of 6 μm thickness. After being fixed with cold acetone for 10 min, the tumor slides were stained with caspase 3 PAb (catalog number: 19677-1-AP) for 1 h, followed by detecting by PV-6001 rabbit kit. The caspase 3 expression was by a confocal microscope at a magnitude of 200×.

## Results and discussion

### Preparation and characterization of Gel conjugation

**Fig. 1:**
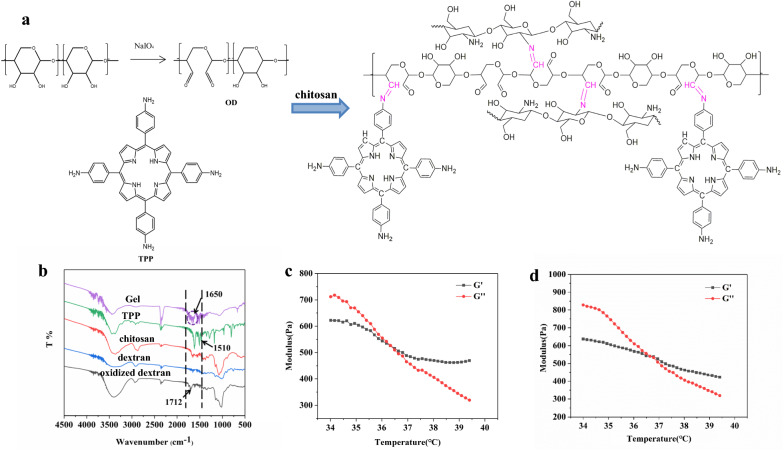
Schematic structural information (**a**), IR spectra (**b**), the storage modulus and loss modulus of OD/TPP hydrogel (**c**) and DOX-CA4P@Gel (**d**)

As shown in Fig. 1a, hydrogel was obtained by a one-step reaction among OD, chitosan and NH_2_-TPP, in which the OD-to-NH_2_-TPP ratio was 1:10, and chitosan (W% = 10%) was to adjust the elasticity. The degree of aldehyde was closely related to the oxidation time by sodium periodate. Considering the reaction time and the degree of aldehyde (Additional file 1: Table S1), the optimum oxidation time was 16 h. The amount of chitosan affected the formation and degradation of hydrogel, results showed in Additional file 1: Table S2 indicated that 73% (*w*_chitosan_%) was optimal for getting fast formation and slowly degradation. As shown in Fig. 1b, the disappearance of characteristic IR absorbance peaks from oxidized dextran (at ~1712 cm^−1^ for C=O) and porphyrin derivative (~1510 cm^−1^ for N–H), and presence of characteristic IR peak from Gel (~1650 cm^−1^ for C=N) [3, 19] confirm the successful reaction between OD and TPP. The gel-forming behavior under physiological conditions was also evaluated by measuring the storage modulus (G′) and loss modulus (G′′), where the sol-gel conversion condition could be determined. As shown in Fig. 1c and d, the conversion occurred at ~36.5 °C for Gel, and ~36.8 °C for DOX-CA4P@Gel, which indicated that loading of CA4P and DOX did not change the gelation process of TPP, chitosan, and OD. Gel could form within 8.8 min, and DOX-CA4P@Gel formed within 8.5 min. Results showed that the hydrogel can be synthesized quickly at a temperature close to physiological temperature, indicating its injectability in vivo.

**Fig. 2:**
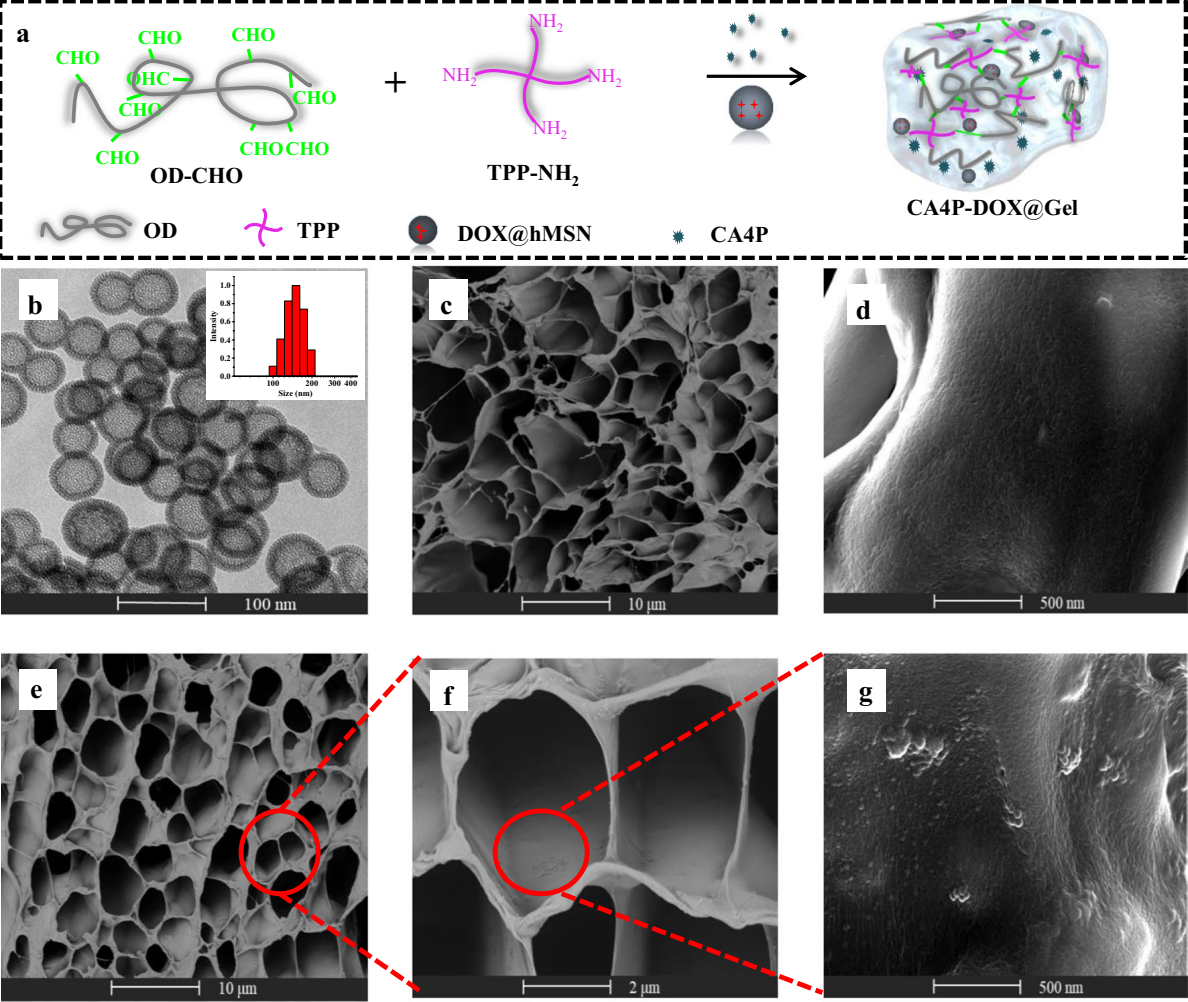
Schematic diagram of DOX-CA4P@Gel (**a**), TEM image (insert: DLS measurement) of hMSN (**b**), SEM images of blank hydrogels (**c**, **d**), and SEM images of DOX-CA4P@Gel (**e**–**g**)

As shown in Fig. 2a, DOX was loaded into hMSN to form DOX@hMSN, and then DOX@hMSN was enveloped by OD/TPP together with CA4P to form dual drugs loaded DOX-CA4P@Gel. The morphology and size distribution histogram of hMSN was shown in Fig. 2b. The average diameter of hMSN was 120 nm from TEM assay, smaller than that measured from DLS (with the average diameter of 142 nm) for hydration effects. The zeta potential analysis was constructed in Additional file 1: Figure S1. Compared to the value of hMSN (− 29.8 ± 1.1 mV), the zeta potentials of hMSN-NH_2_ significantly increased, indicating a modification effect of amino groups. No obvious difference was present between DOX-CA4P@Gel (38.4 ± 3.8 mV) and CA4P@Gel (33.5 ± 2.4 mV). Figure 2c, d was SEM images of the lyophilized cross-section of the hydrogel. Results showed that the hydrogel possessed a three-dimensional network structure with a uniform pore size of about 5 μm. Figure 2e–g showed the three-dimensional network structure of the freeze-dried cross-section of DOX-CA4P@Gel. The results showed that DOX@hMSN did not affect the morphology of the hydrogel network structure, and the bulges in the three-dimensional structure indicated the locations of hMSN. The surface chemical compositions of the hydrogel layers from XPS also confirmed it. Comparing the X-ray photoelectron spectrum of Gel with that of DOX-CA4P@Gel (Additional file 1: Figure S2), the peaks seen at 154.1 eV and 102.5 eV indicate that there were additional Si elements on the surface of Gel, which were resourced from hMSN. According to the absorbance of DOX at 510 nm, where TPP and CA4P didn’t affected its detection (Additional file 1: Figure S3), the drug loading capacity of DOX in hMSN was 633 mg/g, and 52 mg/g for DOX in DOX-CA4P@Gel. The drug loading of CA4P in hydrogel was 12.6 mg/g calculating from HPLC measuring.

### Drug sequential release from DOX-CA4P@Gel

**Fig. 3:**
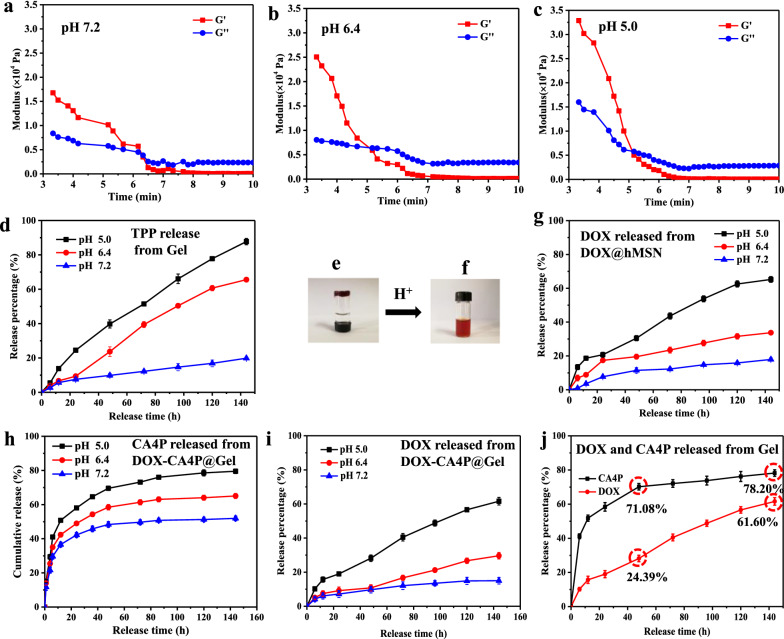
G′ and G′′ of hydrogel in different solution of pH 7.2 (**a**), pH 6.4 (**b**), pH 5.0 (**c**), degradation and release of TPP from hydrogel in PBS with different pH values (**d**), macroscopic state of hydrogel under different acidity conditions (**e**, **f**), drug release of DOX from hMSN under different pH medium (**g**), drug release of CA4P (**h**) and DOX (**i**) from hydrogel in different medium respectively, drug release of CA4P, DOX behavior in pH 5.0 (**j**)

DOX-CA4P@Gel was dispersed in phosphate buffer solution (PBS) solution with different pH values, and the elastic modulus of hydrogel was measured using a rheometer. As shown in Fig. 3a, b and c, the energy storage modulus (G′ = 34,000) of hydrogels at pH of 5.0 was significantly higher than that in pH 7.2 (G′ = 17,000), which proved that the hardness of hydrogels under acidic conditions was more than that in neutral condition, and the hydrogels were more prone to fracture under the same shear force. This result was consistent with the corresponding crushing time (*t*_pH 5.0_=5.0 min, *t*_pH 6.4_=5.2 min, *t*_pH 7.2_=6.4 min). DOX-CA4P@Gel was dispersed in PBS with different pH values, and the degradation of hydrogels was evaluated by measuring the content of decomposed TPP in solution. Results in Fig. 3d showed that the degradation rate of hydrogels was very slow in neutral condition (about 20% at 144 h), while that was significantly increased in weakly acid condition, reaching 65% at 144 h in pH of 6.4. Degradation of hydrogel at pH 5.0 was faster and more than 90% TPP was detected at 144 h. This result testified that pH sensitive degradation of DOX-CA4P@Gel, indicating the application potential as a tumor-selective drug delivery. The images before and after hydrogel degradation were shown in Fig. 3e, f.

CA4P and DOX exhibited pH sensitive and the release profiles were also observed, where faster release rates were shown at acidic condition (pH 5.0) compared with that in neutral condition (Fig. 3h, i). CA4P and DOX have different release behavior. As shown in Fig. 3j, CA4P was rapidly released at the early stage and relatively stable after 48 h, while DOX released slowly firstly and then go fast after 48 h. 59.13 % of CA4P was released from Gel at pH of 6.4 for 48 h, and reached to 63.44% at 144 h (only 5.31% higher than that of 48 h). As shown in Fig. 3g, 68.32% of DOX was release at pH of 5.0 for 144 h but only 16.02% released in a buffer with pH 7.4. This significant release characteristics of CA4P and DOX form Gel were shown in Fig. 3j. In the early stage, the release of CA4P was rapid, reaching 71.08% at 48 h, and slowed down in the later stage, reaching only 78.20% at 144 h. As a contrast, DOX released only 24.39% at 48 h, and increased to 61.60% at 144 h. The degradation of hydrogel in tumor microenvironment was responsible for the early-stage release of CA4P. Furthermore, the hydrogel capping was also gradually removed from sealing the mesoporous of hMSNs under pH of 6.4 and 5.0, which accelerated DOX release because of the protonating of the amine group of DOX.

### Photodynamic performance of hydrogel

**Fig. 4:**
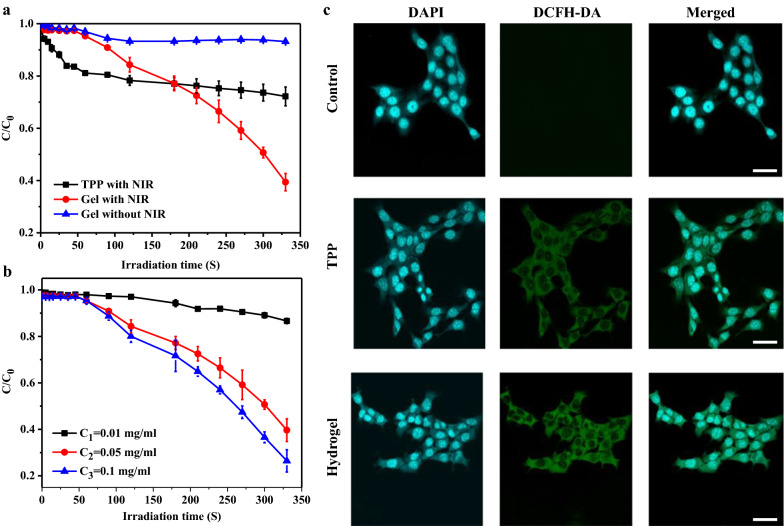
^1^O_2_ generation measurement of hydrogel (**a**), ^1^O_2_ generation depended on concentrations of hydrogel (**b**), ^1^O_2_ generation in cancer cells detected by confocal microscopy (**c**). scale bar: 50 μm

In order to prove that the photosensitivity of porphyrin was not changed significantly after the incorporation into the hydrogel, DPBF was used as the reagent to quantitatively detect the production of singlet oxygen (^1^O_2_) in solution. The results of the irradiation of the samples in the presence of DPBF (absorption monitored at 410 nm) with time are shown in Fig. 4a, where the decrease in DPBF absorption over time for hydrogel compared to TPP, and the decrease was positively correlated with the hydrogel concentrations (as shown in Fig. 4b). With TPP (*φ*_∆(TPP)_ = 0.52) as the control [32], the ^1^O_2_ yield of hydrogel (808 nm, 0.5 W/cm^2^) was 0.91, which may be due to the high viscosity of hydrogel limiting the rotation of porphyrin molecules and avoiding the optical quenching induced by the collision between porphyrin molecules. DCFH-DA was an intracellular singlet oxygen capture agent, and its green fluorescence intensity of cells was directly proportional to the production of ^1^O_2_. The concentration of After incubation of hydrogel with cells, ^1^O_2_ detection was conducted. As shown in Fig. 4c, stronger fluorescence emission as compared with those in TPP was present which was consistent with that in PBS.

**Fig. 5:**
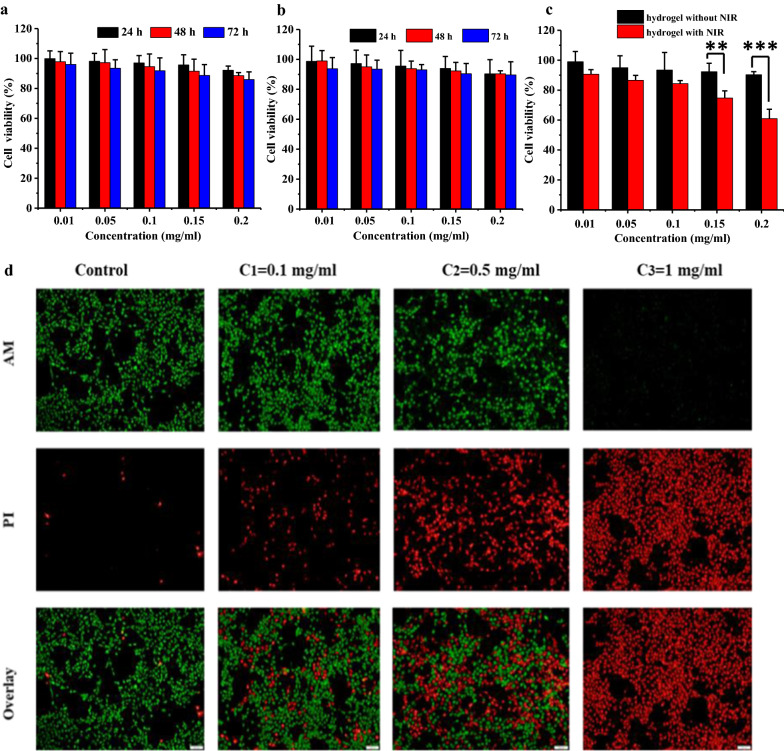
Cell viabilities of L929 (**a**) and 4T1 cells (**b**) after respectively incubation with hydrogel, cell viabilities of 4T1 cells after incubation with hydrogel for 48 h under irradiation (**c**), confocal microscopy imaging of 4T1 cells after incubation with hydrogel under irradiation (red: dead cells, green: live cells) (**d**). Scale bar: 100 nm, *p < 0.05, **p < 0.01

The extracts of blank hydrogel with different concentrations (0.05, 0.1, 0.15 and 0.2 mg/mL) were incubated with L929 and 4T1 cells for 24, 48 and 72 h, respectively. As shown in Fig. 5a, b, the cellular viabilities of L929 and 4T1 cells were above 85%, indicating that the hydrogel had no obvious toxicity to cells without laser irradiation. However, the cellular viability significantly decreased after laser treatment (NIR = 808 nm, 0.5 W/cm^2^, *t *= 5 min). This result shown in Fig. 5c was particularly significant change at high concentration, that is, at 0.2 mg/ml, the cell viability of the hydrogel group without laser assistance was 92.43%, while that of with laser group was reduced to 65.31%, with a significant difference (*p *< 0.05), suggesting that hydrogel has an obvious photodynamic killing effect on 4T1 cells. In order to further intuitively demonstrate the PDT effect of hydrogel on cancer cells, the live/dead cells staining with calcein AM and PI was carried out. The fluorescence imaging results in Fig. 5d indicated that the proportion of dead cells increased significantly with the elevated concentrations of the hydrogel extract. Moreover, with the increase of irradiation time (showed in Additional file 1: Figure S4), the proportion of dead cells also increased significantly, indicating that the photodynamic treatment effect of hydrogel was obvious concentration and time dependent.

### In vitro chemo-photodynamic therapy effect of DOX-CA4P@Gel

Gel, DOX@Gel, CA4P@Gel, and DOX-CA4P@Gel (0.1 mg/ml) were respectively incubated with 4T1 cells for 48 h, and the cell viabilities were measured with or without laser irradiation (808 nm, 0.5 W/cm^2^, 5 min). PBS was used as the control. As showed in Additional file 1: Figure S5, the cell viability of both Gel and DOX@Gel groups were above 90%, indicating that no obvious DOX was released during this period. In contrast, the cell viability of both CA4P@Gel and DOX-CA4P@Gel groups were lower than 70%. Furthermore, cell viability in CA4P@Gel significantly decreased with time lasting to 48 h, indicating that CA4P could be continuously released within 48 h. With laser irradiation, all the groups including Gel+NIR, DOX@Gel+NIR, CA4P@Gel+NIR and DOX-CA4P@Gel+NIR, exhibited enhanced cell killing effect. Especially, the cell activity of DOX-CA4P@Gel+NIR group was lower than 40%, which verified that DOX-CA4P@Gel has significant combinational effect of photodynamic/sequential chemotherapy.

**Fig. 6:**
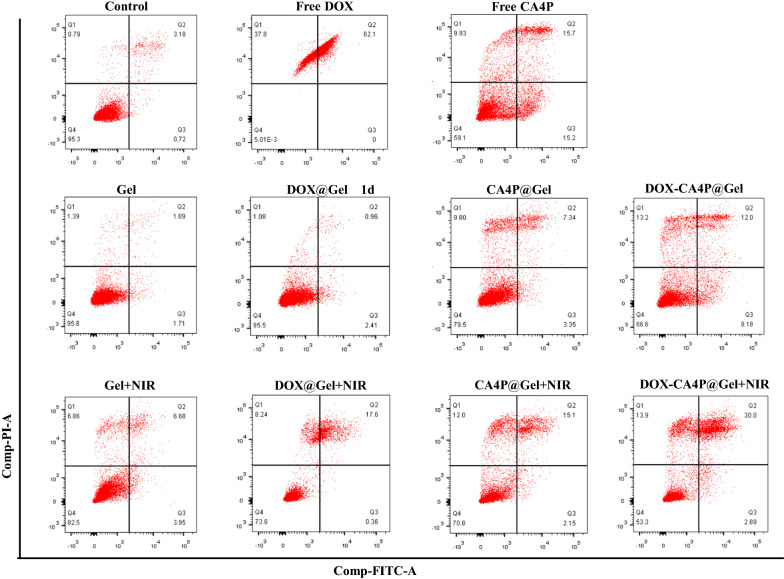
Flow cytometry analysis of 4T1 cells after 24 h of incubation with different groups. PBS was used as the negative control group, and free DOX/CA4P was used as the positive control group (808 nm, 0.5 W/cm^2^, 5 min)

This result was also verified in a flow cytometry assay. As shown in Fig. 6, without laser irradiation, the cell viability (95.5%) of DOX@Gel was not significantly different from that of Gel (95.8%), while CA4P@Gel and DOX-CA4P@Gel decreased to 79.5% and 66.6%, respectively. This is due to DOX-CA4P@Gel mainly showed the release of CA4P within 24 h, while DOX did not show obvious tumor killing effect because of its late release. Furthermore, after the same sample treatment, the cell viability with laser irradiation was significantly lower than that of the non-irradiated group, especially the cell viability of DOX-CA4P@Gel+NIR group decreased from 66.6 to 53.3%, reflecting obvious combined treatment effect.

Comparing the cell viability of groups (longitudinal groups) without laser assistance, results showed that after 24 h of co-incubation with 4T1 cells, only CA4P@Gel (79.5%) and DOX-CA4P@Gel (66.6%) was significantly lower than that of control group (95.3%), while DOX@Gel group was not significantly different from the control group, which proved that early release of CAP4P from DOX-CA4P@Gel was the major reason for cell toxicity before 24 h. Comparing the Gel groups (lateral groups), it was found that the cell viability with laser assistance was significantly lower than that of the without laser assistance group, especially DOX-CA4P@Gel+NIR was only 50%.

### Combinational therapy of DOX-CA4P@Gel

**Fig. 7:**
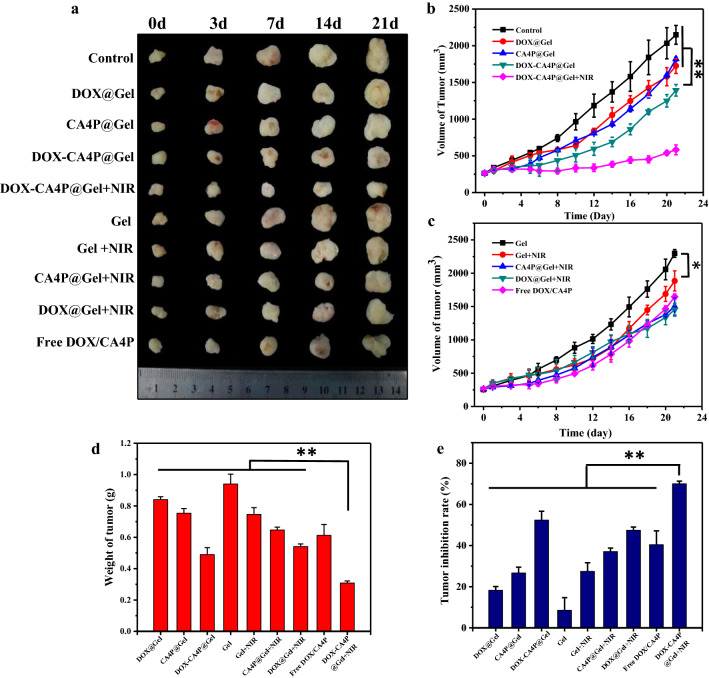
Isolated tumor during the treatment cycle (**a**), tumor volume of the mice during the treatment cycle (**b**, **c**), tumor weight (**d**) and tumor growth inhibition rate (**e**) post-treated by different samples for three weeks. *p < 0.05, **p < 0.01

The 4T1 tumor-bearing mice were respectively treated by a single intratumor injection administration of PBS, Free DOX/CA4P (mixture of 0.4 mg DOX and 1 mg CA4P), Gel, CA4P@Gel, DOX@Gel, DOX-CA4P@Gel, Gel+NIR, CA4P@Gel+NIR, DOX@Gel+NIR, DOX-CA4P@Gel+NIR, where NIR was illuminated for 5 min every other day. The body weight, the volume and weight of the tumor were recorded every day, and the tumor inhibition rate was calculated with PBS as the control group. As can be seen from Fig. 7a–c, the tumor in the CA4P@Gel group grew slowly in the first 6 days and then accelerated, while the tumor in the DOX@Gel group grew rapidly in the first 6 days but slowed down significantly within 6–12 days, which may be explained by the sequential release of CA4P and DOX. With the assistance of laser, the comparison of treatment results between the DOX-CA4P@Gel +NIR group and other groups showed that the tumors in the DOX-CA4P@Gel +NIR group were the smallest and significantly different from those in other groups (*p *< 0.01), indicating that sequential delivery therapy combined with photodynamic therapy had a significant impact on tumor growth. It should be noted that after 18 days of treatment, tumor growth was accelerated in the DOX/CA4P group, with tumor volume greater than that of CA4P-DOX@Gel, which may be due to the fact that CA4P stimulated the formation of tumor vascular collateral after a single dose, thus accelerating tumor growth. In the DOX-CA4P@Gel system, CA4P plays its role first, followed by DOX and PDT. Sequential drug release combined with photodynamic therapy can effectively inhibit tumor growth to a certain extent. The therapeutic effect of each group was also evaluated by measuring tumor weight, and the results were shown in Fig. 7d. The results showed that the tumor weight of the DOX-CA4P@Gel +NIR group was the lowest, which was consistent with that of tumor volume growth curves. As showed in Fig. 7e, the calculated tumor inhibition rate was about 70% for DOX-CA4P@Gel +NIR group, which was significantly different from other groups (*p *< 0.01).

**Fig. 8:**
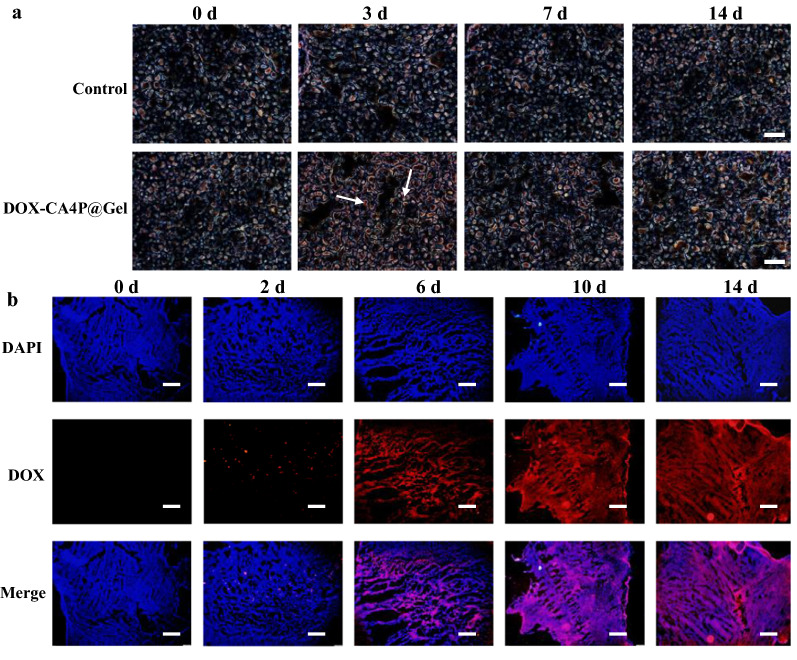
Immunohistochemistry measurement of caspase 3 activity in tumor (**a**), histology of tumors post-injection of DOX-CA4P@Gel. Scale bar: 100 μm

The expression of caspase 3 and DOX fluorescence intensity were used to characterize the location of CA4P and DOX. CA4P can specifically bind to vascular growth factor and prevent its interaction with the receptor, thereby causing apoptosis. The activity of caspase 3 reflects the apoptotic status of tumor cells. At different time points, the caspase 3 activity (apoptotic inducing factor) in the tumors of the DOX-CA4P@Gel group was detected by immunohistochemistry. Results in Fig. 8 showed that compared with that in control group, the caspase 3 expression was significantly up-regulated, reaching the peak on the 3rd day, and gradually went down in the subsequent treatment period. DOX release and accumulation in tumor sections of the DOX-CA4P@Gel group at different time points (2, 6, 10, 14 days) were measured by the fluorescence properties of DOX. The results showed that DOX fluorescence in tumor tissues was extremely weak at the initial stage of treatment (within 2 days), suggesting DOX-CA4P@Gel accumulating in the tumor. With the extension of time (at the 6th days), it retained in tumor for more than 14 d.

### In vivo safety evaluation

To evaluate the possible systemic toxicity, body weigh variation and H&E staining was carried out. Additional file 1: Figure S6 showed that there was no significant statistical difference in the body weight of mice between the control group and each treatment group, indicating that the therapeutic agent had no significant toxicity in vivo. H&E staining of the representative tissue sections, including heart, liver, spleen, lung, and kidney was carried out for the mice treated with PBS and different Gel conjugates at day 21. As shown in Additional file 1: Figure S7, no observable systemic toxicity was noted in all the organs of the tumor-bearing mice treated with the drug-loaded hydrogel groups.

## Conclusions

With biocompatible dextran oxide, chitosan, porphyrin and hMSN as the starting materials, a dual drug carrying system DOX-CA4P@Gel was constructed, which could be injected in tumor and play therapeutic roles in situ. Good biocompatibility DOX-CA4P@Gel was verified by cell tests and H&E staining. Studies on the properties of the materials show that Gel can be degraded slowly under tumor microenvironment, and DOX and CA4P released sequentially for tumor therapy at different time. Animal model experiments showed that DOX-CA4P@Gel+NIR could effectively enhance therapy effect due to the sequential drugs release and photodynamic performance. The DOX-CA4P@Gel constructed in this work is simultaneously injectable, biodegradable, sequentially releasable and photodynamic, providing a feasible route to reduce drug resistance and improve cancer therapy efficiency.

## Supplementary Information

**Additional file 1: Figure S1.** Zeta potential of hMSN, DOX@hMSN-NH2, CA4P@Gel and DOX-CA4P@Gel. **Figure S2.** XPS survey spectra of hMSN, Gel and CA4P-DOX@Gel. ** Figure S3.** Visible spectra of DOX, TPP and CA4P. ** Figure S4.** Fluorescence images of 4T1 cells after incubation with hydrogel with irradiation assistance (red-dead cells, green-live cells). Scale bar: 100 μm. ** Figure S5.** Cell viability of 4T1 cells post different treatment. ***p<0.001. ** Figure S6.** Body weight variation of mice post different treatment. ** Figure S7.** Histolodical analysis of the major organs of mice treated with various drug formulations at day 21 (scale bar is 100 μm). **Table S1.** The degree of aldehyde affected by the oxidation time by sodium periodate. **Table S2.** Effect of chitosan on hydrogel formation and degradation.

## Data Availability

All data analyzed during this study are included in this published article.
