# Supplementary material for: Sequential drug delivery by injectable macroporous hydrogels for combined photodynamic-chemotherapy
Source: J Nanobiotechnology. 2021 Oct 23;19:333. doi: 10.1186/s12951-021-01066-1 (PMC8542336; doi:10.1186/s12951-021-01066-1)
Supplement: Supplementary file 1 — Additional file 1: Figure S1. Zeta potential of hMSN, DOX@hMSN-NH2, CA4P@Gel and DOX-CA4P@Gel. Figure S2. XPS survey spectra of hMSN, Gel and CA4P-DOX@Gel. Figure S3. Visible spectra of DOX, TPP and CA4P. Figure S4. Fluorescence images of 4T1 cells after incubation with hydrogel with irradiation assistance (red-dead cells, green-live cells). Scale bar: 100 μm. Figure S5. Cell viability of 4T1 cells post different treatment. ***p<0.001. Figure S6. Body weight variation of mice post different treatment. Figure S7. Histolodical analysis of the major organs of mice treated with various drug formulations at day 21 (scale bar is 100 μm). Table S1. The degree of aldehyde affected by the oxidation time by sodium periodate. Table S2. Effect of chitosan on hydrogel formation and degradation. [file 12951_2021_1066_MOESM1_ESM.docx]

Supporting information

**Sequential drug delivery by injectable macroporous hydrogels for combined photodynamic-chemotherapy**

Yuanyuan Zhong^a†^, Li Zhang^a†^, Shian Sun^c^, Zhenghao Zhou^a^, Yunsu Ma^a^, Hao Hong^b^*, Dongzhi Yang^a^*

^a^ Jiangsu Key Laboratory of New Drug Research and Clinical Pharmacy, Xuzhou Medical University, Xuzhou, Jiangsu 221004, China

^b^ Medical School, Nanjing University, Nanjing, Jiangsu 210093, China

^c^ Xuzhou Air Force College, Xuzhou, Jiangsu 221000, China

† These authors contributed equally to this work.

*Corresponding author:

Dongzhi Yang (dongzhiy@xzhmu.edu.cn)

Hao Hong (haohong@nju.edu.cn)

Table S1 The degree of aldehyde affected by the oxidation time by sodium periodate

| Oxidation time (h) | The degree of aldehyde (%) |
| --- | --- |
| 1.5 | 19.3 |
| 3 | 19.7 |
| 6 | 28.1 |
| 8 | 39.8 |
| 16 | 65.6 |
| 24 | 63.7 |

Table S2 Effect of chitosan on hydrogel formation and degradation

| *w*_chitosan_  (%) | Hydrogel formation time  (s) | Degradation of hydrogel at 6 h (%) |
| --- | --- | --- |
| 84 | 0 | 11.6 |
| 80 | 0 | 7.7 |
| 77 | 132 | 6.9 |
| 73 | 37 | 6.6 |
| 70 | 85 | 2.3 |
| 67 | - | 0 |
| 65 | - | 0 |

"^-^" was that the hydrogel was not formed.

Figure S1 Zeta potential of hMSN, DOX@hMSN-NH_2_, CA4P@Gel and DOX-CA4P@Gel.

Figure S2 XPS survey spectra of hMSN, Gel and CA4P-DOX@Gel.

Figure S3 Visible spectra of DOX, TPP and CA4P.


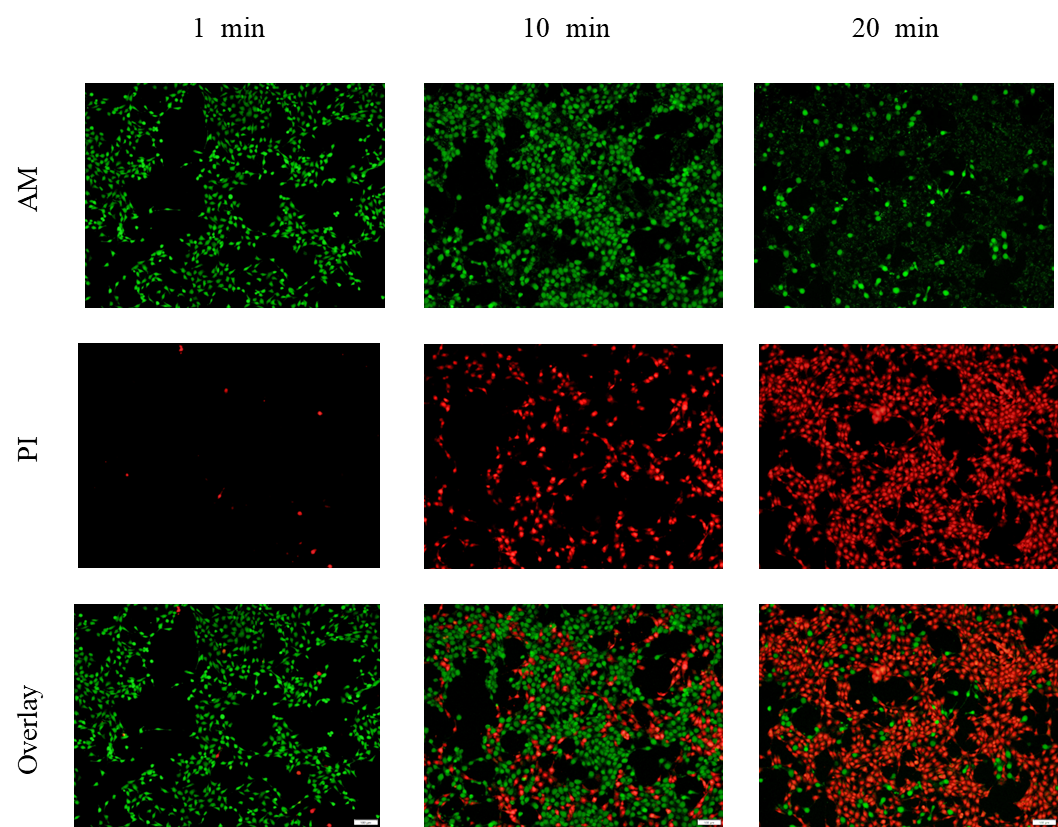


Figure S4 Fluorescence images of 4T1 cells after incubation with hydrogel with irradiation assistance (red-dead cells, green-live cells). Scale bar: 100 μm.

Figure S5 Cell viability of 4T1 cells post different treatment. ****p*<0.001.

Figure S6 Body weight variation of mice post different treatment.

Figure S7 Histolodical analysis of the major organs of mice treated with various drug formulations at day 21 (scale bar is 100 μm)
